# 

**Published:** 1915-07-10

**Authors:** 


### Doctors' Wills.

Mr. George McKerrow, aged sixty-five, of Ayr, late resident physician and resident surgeon, Royal Infirmary, Glasgow, surgeon to the Ayr County Hospital, clerk to the Eye Infirmary and to the Skin and Ear Dispensary, Glasgow, has left personal estate of the total value of £32,956.

Mr. Daniel Carmichael, F.R.C.S., L.R.C.P., of Newcastle-on-Tyne, lately medical officer of health for Bedlington, who died on February 28, has left estate valued at £8,857 gross, with net personalty £8,060. The testator, after creating two life interests, left, on the death of the survivor, the ultimate residue of his estate, after the payment of two small legacies, equally between two Presbyterian charities.

### Medical Appointments.

Dr. John Beattie Dunlop, M.A. Cantab., has been appointed by the board of management of the Bradford Royal Infirmary to the post of honorary physician, rendered vacant by the retirement of Dr. Thomas Wilmot. Educated at Cambridge and Edinburgh Universities, Dr. Dunlop qualified as M.R.C.S., L.R.C.P. Lond. in 1901, and in 1904 graduated M.B., B.C. Cantab. He has acted as honorary assistant physician to the Royal Infirmary since 1906.

### Gifts and Bequests.

MR. ROBERT WILLIAM WEBB, of Godalming, Surrey, has left £300 to the Royal Surrey County Hospital.

MR. FENWICK CARRE, of Letterkenny, County Donegal, surgeon and physician, who died on April 2, left personal property in the United Kingdom valued at £7,993. The testator left £100 to the Royal Medical Benevolent Institution at Dublin, besides several other legacies to charity.

THE Chelsea Hospital for Women, which is giving special facilities for the admission of the wives and near relatives of soldiers at the Front and also to Belgian refugees, has received a further sum of £1,000 from the executors of the T. S. Whitaker estate towards its rebuilding fund.

During the absence on military service of the medical superintendent and the assistant medical officer of the London County Council's Epileptic Colony, arrangements have been made for Dr. S. C. Elgee, the first assistant medical officer at Colney Hatch Mental Hospital, to take charge of the colony as acting medical superintendent.

---

The directors of the London County and Westminster Bank, Ltd., have declared, we understand, an interim dividend of 9 per cent. for the half-year ending June 30. The dividend, 9s. per share (less income-tax), will be payable on August 3.

RATES OF SUBSCRIPTION: Twelve months, 6/6; Six months, 4/-; Three months, 2/-, post free to any address in the United Kingdom. Abroad, Twelve months, 8/8; Six months, 5/-; Three months, 2/6, post free.—Address The Subscription Dept., "THE HOSPITAL," The Hospital Building, 28/29 Southampton Street, Strand, London, W.C.

